# AMPK/mTOR Signaling in Autophagy Regulation During Cisplatin-Induced Acute Kidney Injury

**DOI:** 10.3389/fphys.2020.619730

**Published:** 2020-12-17

**Authors:** Ying Wang, Zhiwen Liu, Shaoqun Shu, Juan Cai, Chengyuan Tang, Zheng Dong

**Affiliations:** ^1^Department of Nephrology, The Second Xiangya Hospital at Central South University, Changsha, China; ^2^Department of Cellular Biology and Anatomy, Charlie Norwood Veterans Affair Medical Center, Medical College of Georgia, Augusta University, Augusta, GA, United States

**Keywords:** cisplatin, acute kidney injury, nephrotoxicity, autophagy, AMPK, mTOR

## Abstract

Autophagy is a conserved, multistep pathway that degrades and recycles dysfunctional organelles and macromolecules to maintain cellular homeostasis. Mammalian target of rapamycin (mTOR) and adenosine-monophosphate activated-protein kinase (AMPK) are major negative and positive regulators of autophagy, respectively. In cisplatin-induced acute kidney injury (AKI) or nephrotoxicity, autophagy is rapidly induced in renal tubular epithelial cells and acts as a cytoprotective mechanism for cell survival. Both mTOR and AMPK have been implicated in the regulation of autophagy in cisplatin-induced AKI. Targeting mTOR and/or AMPK may offer effective strategies for kidney protection during cisplatin-mediated chemotherapy.

## Introduction

Macroautophagy, referred to hereafter simply as autophagy, is a process of “self-eating” in cells that contributes to the resolving and recycling of various cytoplasmic components by the lysosomal degradation pathway (Feng et al., 2014; Kaushal and Shah, 2016). The occurrence of autophagy involves the core autophagy machinery consisting of a large group of autophagy-related (ATG) proteins (Mizushima et al., 2011; Kim et al., 2018). Mammalian target of rapamycin (mTOR) and adenosine-monophosphate activated-protein kinase (AMPK) are major regulators of autophagy. mTOR negative modulates autophagy by phosphorylating the Atg1/unc-51 like autophagy activating kinase 1 (ULK1) to prevent the initiation of autophagy (Alers et al., 2012). AMPK positively regulates autophagy via suppressing mTOR complex 1 (mTORC1) activity in both direct and indirect manners (Inoki et al., 2012; Ji et al., 2015). Moreover, AMPK can bind, phosphorylate, and then directly activate ULK1/2 to induce autophagy (Kim et al., 2011; Yang et al., 2018). Thus, targeting AMPK and mTOR signaling represents therapeutic strategies of autophagy-related diseases, including cardio vascular diseases, ischemia-reperfusion injury, diabetic complications, and so on.

Acute kidney injury (AKI) is a major kidney disease characterized by a rapid loss of renal functions (Kellum and Prowle, 2018). Nephrotoxicity, renal ischemia-reperfusion and sepsis are major clinical causes of AKI (Bucaloiu et al., 2012; Yang et al., 2015; Mei et al., 2016). Cisplatin is one of the most potent chemotherapeutic drugs for cancer treatment (Wang and Lippard, 2005). However, the use of cisplatin is limited by its side effects in normal tissues, especially nephrotoxicity (Pabla and Dong, 2008; Zhang et al., 2017; Wang et al., 2018; Xu et al., 2020). The pathogenesis of cisplatin-induced AKI is complex and multifactorial. Recent studies have demonstrated autophagy activation and its kidney protective role in experimental models of cisplatin-induced AKI (Kaushal, 2012; Kimura et al., 2012; Zhang et al., 2017). Here, we summarize the current understanding of autophagy in cisplatin-induced AKI with a focus on the regulation by mTOR and AMPK.

## Autophagy in Cisplatin-Induced AKI

**FIGURE 1:**
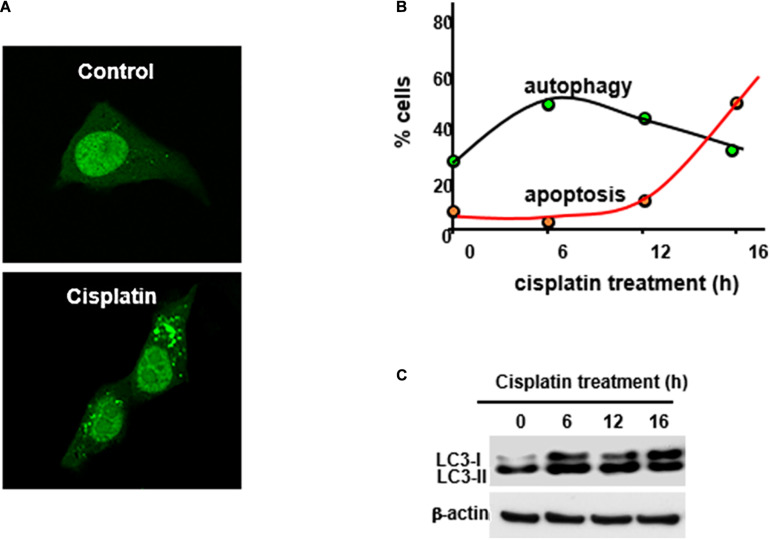
Cisplatin-induced autophagy in kidney proximal tubular cells. Rat kidney proximal tubular cells transiently transfected with GFP-LC3 were incubated with 20 μM cisplatin for 0–16 h to record GFP-LC3 signal **(A)**, assess% cells with punctate/autophagic GFP-LC3 staining and % cells with apoptotic morphology **(B)**, and analyze LC3-II formation by immunpblottinh **(C)**. This figure was modified from Periyasamy-Thandavan et al. (2008).

The first evidence of autophagy activation in cisplatin-induced AKI was demonstrated in 2008 by two independent studies (Periyasamy-Thandavan et al., 2008; Yang et al., 2008). We observed the increase of autophagosome formation and light chain 3 II (LC3-II) accumulation in cultured renal proximal tubular cells following cisplatin exposure. Time course study showed that autophagy occurred before caspase activation and apoptosis in these cells (Figure 1). Importantly, blockade of autophagy by pharmacological inhibitors or autophagy-gene knockdown increased renal tubular cell apoptosis during cisplatin treatment, suggesting a protective role of autophagy (Ji et al., 2015). These observations were verified by Kaushal and colleagues (Yang et al., 2018). Interestingly, a later study showed that autophagy was dramatically induced by 10 μM of cisplatin, whereas a higher dose (50 μM) of cisplatin induced mainly apoptosis and not autophagy (Rovetta et al., 2012). These findings suggest that autophagy is an adaptive and defense mechanism against cisplatin-induced renal tubular cell death or nephrotoxicity. Induction of autophagy in renal tubular cells *in vivo* was also verified. In 2008, we observed the accumulation of autophagic vesicles in renal proximal tubulars cells by electron microscopy in C57BL/6 mice subjected to cisplatin treatment (Periyasamy-Thandavan et al., 2008). Consistently, later studies showed an increase of autophagosome formation in renal tubular cells during cisplatin treatment of GFP-LC3 autophagy reporter mice (Inoue et al., 2010; Takahashi et al., 2012). Manipulation of autophagy by using genetic and/or pharmacologic approaches has provided further evidence for a critical role of autophagy in cisplatin nephrotoxicity. Primary cultures of proximal tubular epithelial cells from proximal tubule-specific Atg7-konckout (KO) mice were shown to be more susceptible to cisplatin-induced apoptosis in comparison with the cells from wild-type mice littermates (Jiang et al., 2012). *In vivo*, inhibition of autophagy by chloroquine was shown to exacerbate cisplatin-induced tubular injury and renal function loss, while activation of autophagy by rapamycin was shown to attenuate tubular injury and protect against cisplatin-induced AKI in mice (Herzog et al., 2012; Jiang et al., 2012). Moreover, proximal tubule-specific deletion of Atg5 or Atg7 increase the sensitivity of mice to cisplatin-induced AKI (Jiang et al., 2012; Takahashi et al., 2012). Mechanistically, activation of p53, apoptosis, DNA damage and c-Jun N terminal kinase appeared to contribute to more severe kidney injury in these Atg-deficient mice (Jiang et al., 2012). Taken together, autophagy is an important protective mechanism that can up-regulate for kidney protection during cisplatin-induced AKI.

## AMPK and mTOR Signaling

**FIGURE 2:**
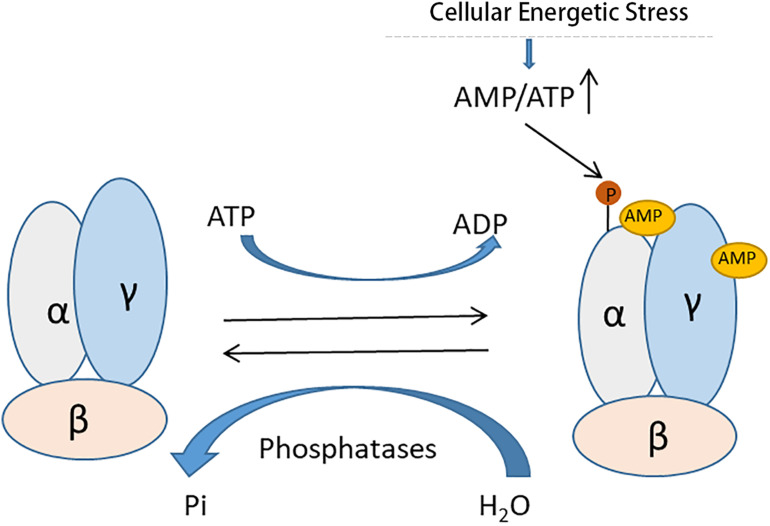
Allosteric activation of AMPK by AMP. AMPK is a heterotrimeric protein complex consisting of α, β, and γ subunits. Under conditions of cellular energetic stress, increased AMP binds the γ subunit of AMPK results in phosphorylation of the catalytic α subunit for its activation.

AMPK, originally discovered by the Carbson and his colleagues in 1973 (Li et al., 2017) is a pivotal regulator of several defensive molecules in various pathological processes. As a serine/threonine kinase, AMPK exists ubiquitously in a variety of cells and tissues as a hetero-trimer of α, β, γ subunits (Jiang et al., 2017). AMPK activity is mainly regulated through a direct allosteric activation by adenosine monophosphate (AMP) at the γ subunit that results in phosphorylation of the catalytic α subunit (Hardie, 2004; Ix and Sharma, 2010; Figure 2). Under conditions of cellular energy shortage, such as starvation and related cell stress, an increase of the ratio of AMP/adenosine triphosphate (ATP) activates AMPK for maintaining energy homeostasis. AMPK can activate a great number of catabolic processes in multicellular organisms such as glucose uptake and metabolism, while simultaneously suppressing several anabolic pathways, such as protein, lipid and carbohydrate biosynthesis and AMPK may also take part in the regulation of autophagy induction (Alers et al., 2012). Meanwhile, Recent studies have demonstrated that activation of AMPK signaling is protective in disease conditions, including AKI (Liu et al., 2018), heart diseases (Gulati et al., 2013), liver diseases (Mann et al., 2016), lung diseases (Schaffer et al., 2015) and so on. For instance, emerging evidence suggested that activation of AMPK was renoprotective in renal ischemia- reperfusion-, nephrotoxin- and sepsis-induced AKI (Pan et al., 2015; Yadav et al., 2015; Fan et al., 2018).

mTOR is also a serine/threonine kinase, which functions as a central regulator of cell growth, proliferation and survival in response to nutritional status, growth factors, and stress signals. There are two mTOR complexes, including mTORC1 and mTORC2. mTORC1, which is sensitive to inhibition by rapamycin, integrates a variety of signals (e.g., growth factors, amino acids, cellular energy content, and cellular stress) to regulate cellular activities like protein synthesis, energy metabolism, and stress response. mTORC2, which is insensitive to rapamycin, seems to be activated by insulin and related pathways, and controls several downstream AGC kinases through their hydrophobic motif phosphorylation contributing to cell survival and cytoskeletal organization (Raught et al., 2001; Grahammer et al., 2017; Vizza et al., 2018). Accumulating evidence suggests that mTOR has an important effect on renal cell homeostasis and autophagy. As such, it plays a regulatory role in the pathogenesis of AKI, glomerular disease, polycystic kidney disease (PKD), and renal transplant rejection (Fantus et al., 2016).

mTORC1 has long been recognized as an essential negative regulator of autophagy. Mechanistically, mTORC1 inhibits autophagy mainly by phosphorylating ULK1 (Atg1 in yeast) to maintain it in an inactive state, and thereby inhibiting autophagy initiation (Hosokawa et al., 2009). Moreover, recent studies also showed that mTORC1 may also suppress autophagy by directly phosphorylating activating molecule in beclin-1-regulated autophagy (AMBRA1), a component of the class III phosphoinositide 3-kinase (VPS34)–Beclin1 (ATG6) complex that recruits downstream effectors to the site for nucleation of autophagosomes (Nazio et al., 2013). In contrast to mTORC1, AMPK acts as a positive regulator of autophagy that directly phosphorylates ULK1 to induce autophagy (Kim et al., 2011; Laker et al., 2017). Interestingly, there may be a crosstalk between mTORC1 and AMPK in autophagy regulation. For instance, Kim et al. showed that ULK1 can stably bind AMPK, and this interaction is inhibited by the mTORC1-dependent ULK1 phosphorylation (Kim et al., 2011). Consistently, the interaction between ULK1 and AMPK, and ULK1 phosphorylation by AMPK, are increased when mTOR is inhibited by rapamycin. The complex crosstalk and feedback of these three interconnected proteins may further fine-tune the autophagic response under metabolic stress conditions (Kim et al., 2011). In addition, AMPK may crosstalk with mTOR signaling by phosphorylate tuberous sclerosis complex 1/2 (TSC1/2) and RAPTOR (upstream regulator and critical component of mTORC1) for autophagy activation (Alers et al., 2012).

## Upstream Regulatory Molecules of AMPK or mTOR

AMPK can be phosphorylated and activated by a variety of upstream kinases, including calcium/calmodulin-dependent protein kinase 2 (CAMKK2), serine/threonine protein kinase STK11, and mitogen-activated protein kinase kinase 7 (TAK1) (Shaw et al., 2004; Anderson et al., 2008; Herrero-Martín et al., 2009). Upon activation, AMPK can directly phosphorylates serine/threonine protein kinase ULK1 to induce autophagy and/or phosphorylates hamartin–tuberin and regulatory-related proteins of mTOR (RAPTOR), one of the components of mTORC1, thereby inhibiting mTORC1 for autophagy activation (Gwinn et al., 2008; Lee et al., 2010). In contrast, mTOR is activated by amino acids through the RAG family of GTPAases (Mossmann et al., 2018). mTOR can also be activated by growth factors via protein kinases like RACα serine/threonine protein kinase (AKT) and mitogen-activated protein kinase kinase (MAPKK)-extracellular signal-regulated kinase (ERK) (Tang et al., 2020).

## AMPK and mTOR in Autophagy During Cisplatin Nephrotoxicity

**FIGURE 3:**
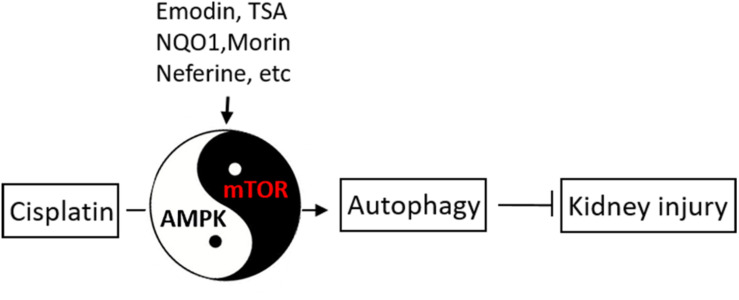
AMPK and mTOR in autophagy regulation during cisplatin-induced kidney injury. Upon cisplatin exposure, autophagy is induced rapidly in renal tubular cells to protect against kidney injury. AMPK and mTOR may play opposite, regulatory roles in autophagy induction by cisplatin. Several pharmacological agents may stimulate autophagy through AMPK/mTOR to afford kidney protection.

Recent studies have indicated regulatory roles of AMPK and mTOR pathways in cisplatin-induced AKI (Figure 3). In 2016, Liu et al. (2016) showed that emodin (1,3,8-trihydroxy-6-methylanthraquinone, a natural anthraquinone) significantly ameliorated cisplatin-induced damage and apoptosis in rat renal tubular cells, and notably, this effect was abolished by bafilomycin A1 (an autophagy inhibitor), suggesting a role of autophagy in the protective effect of emodin. Mechanistically, Emodin was shown to induce the phosphorylation and activation of AMPK, whereas decreasing mTOR activation. Moreover, pharmacological inhibitors of AMPK abolished not only emodin-induced autophagy activation, but also its anti-apoptotic effect, supporting a critical role of AMPK in the effects of emodin (Liu et al., 2016). Kim and colleagues (Liu et al., 2018) further showed that knockdown of NAD(P)H: quinoneoxidoreductase 1 (NQO1, a highly inducible cytoprotective gene) resulted in a marked increase of autophagosomes in cisplatin-treated cells, which was accompanied by reactive oxygen species (ROS) increase. *In vivo*, NQO1-KO mice had increases in the expression of autophagy-associated proteins. Furthermore, silencing of NQO1 enhanced the effect of rapamycin and led to TSC2 phosphorylation via AMPK, triggering autophagy cascade. These results suggest that NQO1 induces autophagy in cisplatin-induced AKI via the AMPK/TSC2/mTOR signaling pathway (Kim et al., 2016). Neferine, a bisbenzylisoquinoline alkaloid, is used as an ingredient in soup and tea (Li et al., 2017). Li et al. (2017) showed that neferine induced autophagy to protect against cisplatin-induced renal tubular cell injury through the activation of the AMPK. In addition, a recent study by Singh et al. found that morin (a natural flavonoid) increased autophagy by increasing the phosphorylation and activation of AMPK and diminishing the activation of mTOR, leading to reduced ROS generation, nuclear DNA damage, inflammatory activity, and apoptosis (Singh et al., 2018). Our latest study (Liu et al., 2018) demonstrated that the autophagy-enhancing effect of histone deacetylase (HDAC) inhibitors (e.g., trichostatin A or TSA). TSA enhanced autophagy during cisplatin treatment of renal tubular cells, which was accompanied with AMPK activation and marginal inactivation of mTOR as indicated by decrease in phosphorylated p70 ribosomal protein S6 kinase (p-P70S6K). Similar effects of TSA were shown *in vivo* in mouse kidneys, further indicating that TSA may enhance autophagy in renal tubular cells to protect kidneys by activating AMPK and suppressing mTOR during cisplatin-induced AKI (Liu et al., 2018). Ginsenoside Rb3 (G-Rb3) is one of protopanaxadiol triterpenoid saponin (Xing et al., 2019). Xing et al. (2019) demonstrated that G-Rb3 protected against renal tubular cell injury through the regulation of AMPK/mTOR-mediated autophagy and inhibition of apoptosis during cisplatin nephrotoxicity both *in vitro* and *in vivo* models. Furthermore, a recent study by Lee et al. (2020) showed that 3-dehydroxyceanothetric acid 2-methyl ester (3DC2ME, isolated from roots of jujube) inhibited the AMPK/mTOR-mediated signaling pathway involved in autophagy regulation to enhance the cellular protective effect of 3DC2ME, thereby relieving cellular apoptosis in cisplatin nephrotoxicity in pig kidney epithelial cell line (LLC-PK1 cells). Together, these findings demonstrate that AMPK/mTOR-mediated autophagy plays an important role in cisplatin nephrotoxicity.

## Targeting mTOR for Therapy in Cisplatin Nephrotoxicity

mTOR has been reported to play a complicated role in various pathophysiological aspects of cisplatin-induced AKI, such as increase of oxidative stress, proximal tubule injury, and renal dysfunction. There is increasing evidence showing that mTOR signaling contributes to the pathogenesis of a variety of kidney diseases. Particularly, mTOR activation is frequently found in cisplatin-induced AKI, contributing to delayed renal function deterioration. Pertaining to autophagy, we recently monitored mTOR phosphorylation on Ser2448 to analyzing the status of mTOR activation during cisplatin-induced AKI (Zhang et al., 2017). Cisplatin induced a significant phosphorylation of mTOR-Ser2448, which was accompanied by phosphorylation of p70S6 kinase, a well-established downstream target of mTOR. Rapamycin blocked p70S6 kinase phosphorylation and enhanced autophagy during cisplatin treatment of renal tubular cells. These findings revealed that mTOR activation negatively regulates autophagy in cisplatin-induced AKI (Zhang et al., 2017). We further determined a regulatory role of protein kinase B (AKT) on mTOR and autophagy. VIII, a pharmacologic inhibitor of AKT, suppressed mTOR as indicated by the attenuation of p70S6K phosphorylation, and augmented autophagy during cisplatin treatment. Inhibition of AKT decreased mTOR activation and increased autophagy, demonstrating that AKT may act upstream of mTOR to quench autophagy during cisplatin treatment. Further work showed that protein kinase Cδ (PKCδ) may directly phosphorylate and activate AKT for mTOR activation, which then induces inhibitory phosphorylation of ULK1, resulting in autophagy inhibition. As a result, this study delineated the PKCδ-AKT-mTOR-ULK1 signaling pathway that is activated to negatively control autophagy in renal tubular cells during cisplatin treatment, shedding new light on autophagy and kidney cell death regulation in cisplatin-induced AKI (Zhang et al., 2017). On the other hand, AKT-mTOR consist a pivotal pathway for protein synthesis that is essential for cell proliferation, drug resistance, and viability (Ruggero and Sonenberg, 2005). They also play an important role in the maintenance of renal function in cisplatin nephrotoxicity. During cisplatin treatment, inhibition of mTOR with rapamycin decreased the expression of death-associated protein 5 (DAP5/p97) and B-cell lymphoma-2 (Bcl-2) in human kidney tubular cells. These findings suggest that the mTOR functions as a positive regulator of cell survival during cisplatin treatment of renal tubular cells (Gao et al., 2012). Huaier polysaccharide (HP-1), an extraction of Trametes robiniophila Murr, relieved the expression of oxidative stress, inflammation and mitochondrial dysfunction, thereby protecting against kidney injury in cisplatin-treated mice (Fang et al., 2019). In human proximal tubule epithelial cell line (HK–2), HP inhibited cell apoptosis and cell cycle arrest by reducing the expression of phosphatidylinositol 3-kinase (PI3K), AKT and mTOR during cisplatin treatment, resulting in attenuating cisplatin nephrotoxicity (Fang et al., 2019). Chen et al. (2020) indicated that the inhibition of the enhancer of zeste homolog 2 (EZH2) upregulated Deptor level, subsequently suppressed mTORC1 and mTORC2 activities, decreased HuR and Bcl-2 expression, leading to apoptosis in renal tubular cells during cisplatin treatment in NRK-52E cells. In this regard, up-regulation of mTOR may afford kidney protection. For example, curcumin has been found to significantly attenuate cisplatin-induced AKI through regulating mTOR and its downstream molecules (p70S6K1, p-eukaryotic translation initiation factor 4E (eIF4E)-binding protein 1 or p-4E-BP1 and p-Akt) in kidney (Sahin et al., 2014). In addition, Zuo’s laboratory (Yang et al., 2019) demonstrated that miR-199a-3p directly bound to mechanistic target of the 3′-untranslated region of mTOR, and miR-199a-3p overexpression decreased the expression and phosphorylation of mTOR in cisplatin-treated HK2 cells. They further verified that p53 suppressed mTOR induction by activating miR-199a-3p under this condition, might providing a potential therapeutic target of AKI (Yang et al., 2019). Together, these studies indicate that mTOR generally plays a protective role in cisplatin nephrotoxicity.

## Targeting AMPK for Therapy in Cisplatin Nephrotoxicity

AMPK is generally recognized as a sensor of cellular energetic status. When cellular energy is low, AMPK is activated to increase nutrient (e.g., glucose, fatty acid) uptake and usage. In kidneys, AMPK is known to plays a pivotal role in normal renal physiology and the pathogenesis of renal diseases, including cisplatin-induced AKI. In 2015, Wei et al. (2015) demonstrated that AMPK is activated in the kidney in cisplatin-treated mice. Moreover, inhibition of AMPK by using si-*ampk* or pharmacological inhibitors (compound C) suppressed autophagy in primary kidney cells after cisplatin treatment, leading to more severe DNA damage and cisplatin-induced AKI. Together, these finding revealed that AMPK-regulated autophagy played an important role in cisplatin nephrotoxicity (Wei et al., 2015). Morigi et al. (2015) revealed that administration of AICAR (5-aminoimidazole-4-carboxamide ribonucleotide, an activator of AMPK) could restore sirtuin 3 (Sirt3) to improve mitochondrial dynamics during cisplatin treatment and protects against AKI. Apparently, the restoration of Sirt3 by AICAR prevented the accumulation of dynamin-related protein-1 (DRP1) to mitochondria and consequent mitochondrial fragmentation, providing insightful information about the protective function of AMPK during cisplatin-induced AKI (Morigi et al., 2015). In 2019, Tsogbadrakh et al. (2019) further proved that AICAR augmented the expression of p-AMPK to protect against cisplatin-induced acute tubular injury in NRK-52E cells and in mice. Moreover, AICAR markedly decreased Janus kinase 2 (JAK2) and signal transducer and activator of transcription 1 (STAT1) expression and increased suppressor of cytokine signaling 1 (SOCS1) expression under this condition. Together, this study suggested that the protective effect of the AMPK activator AICAR might alleviate cisplatin-induced AKI through JAK2/STAT1/SOCS1 pathway (Tsogbadrakh et al., 2019). In 2016, Zhang et al. (2016) showed that pioglitazone, a PPAR-γ agonist, could ameliorate cisplatin nephrotoxicity and improve renal function in mice, and interestingly, pioglitazone may do so by activating AMPK. Mechanistically, AMPK activation by pioglitazone increased the expression and activation of Sirt1 and its binding of NF-kB p65, resulting in robust decreases of p65 acetylation, nuclear translocation and NF-kB-related gene transcription. Pioglitazone also decreased the expression of NF-kB during cisplatin treatment. These results suggest that AMPK may protect kidneys during cisplatin-induced AKI by attenuating NF-kB-associated inflammation via Sirt1-mediated acetylation of p65 (Zhang et al., 2016). Metformin, a common used antidiabetic drug, is also a well-known AMPK stimulator. In 2016, Li et al. (2016) demonstrated the beneficial effect of metformin in a rat model of cisplatin-induced AKI. In rat kidney tubular NRK-52E cells, Metformin activated AMPK to up-regulate autophagy for cell protection during cisplatin treatment. The protective effect of metformin was abolished by inhibitors of AMPK or autophagy. *In vivo*, pretreatment of metformin also enhanced both AMPK and autophagy and protected against cisplatin-induced AKI in rats, further supporting a critical role of AMPK-mediated autophagy in the effect of metformin (Li et al., 2016). Maltol (3-hydroxy-2-methyl-4-pyrone), a by-product of the maillard reaction in starch and sucrose pyrolysis, is known as the safe and reliable food preservative and natural antioxidant (Mi et al., 2018). Mi et al. (2018) suggested that maltol restored the decreased of PI3K/Akt and mTOR levels by cisplatin by increasing the expression of AMPK following cisplatin treatment in HEK293 cells. In addition, maltol inhibited B-cell lymphoma 2-associated X (Bax) and caspase 3 expressions through suppressing the activity of p53 under this condition. As such, maltol play a protective role in cisplatin nephrotoxicity through AMPK/PI3K/AKT and p53 pathways (Mi et al., 2018). Lithium is a validated autophagy inducer with potent efficacy (Bao et al., 2019). Bao et al. (2019) suggested that Lithium protect renal tubular cells against cisplatin nephroxicity. Lithium activated and phosphorylated AMPKα to enhance autophagy to protect cell following cisplatin treatment both *in vitro* and *in vivo.* Under this condition, compound C, inhibition of AMPKα, remarkably abrogated lithium-induced autophagy, and reduced the protective role of lithium (Bao et al., 2019). In addition of AKI, AMPK has been implicated in other kidney diseases, including diabetic kidney disease (DKD). For instance, Resveratrol was shown to enhance AMPK activation to reduce extracellular matrix (ECM) expansion, inflammation and proteinuria in db/db type 2 diabetic mice (Kim et al., 2013). Both metformin and AICAR effectively mitigate streptozotocin (STZ)-induced diabetic kidney injury *in vivo* and high glucose-induced tubular hypertrophy by activating AMPK and suppressing mTOR (Lee et al., 2007; Sokolovska et al., 2010). A recent study further demonstrated that Berberine could activate AMPK and autophagy to ameliorate high glucose-induced apoptosis in podocytes (Sokolovska et al., 2010). More recently, Jin et al. (2020) demonstrated that AMPK activation (Thr172 phosphorylation) which is associated with p53 phosphorylation in cisplatin-treated cultured renal tubular epithelial cells. silencing AMPK by siRNA reduced p53 phosphorylation, Bax induction, and caspase 3 activation after cisplatin treatment. Furthermore, compound C inhibited AMPK activation, p53 phosphorylation, and apoptosis activation in cell and mouse model. Together, these findings proved a protective role of AMPK-p53-Bax signaling pathway in cisplatin-induced tubular epithelial cell apoptosis (Jin et al., 2020). Together, these results suggest that activation of AMPK may enhance autophagy and alleviate the development of DKD. Therefore, AMPK may be a potent therapeutic target in both acute and chronic kidney diseases.

## Targeting AMPK and/or mTOR for Therapy in Cisplatin-Induced Fibrosis

Some injured renal tubules have the ability of regeneration, but renal tubular repair after severe or occasional AKI is often incomplete, leading to renal interstitial fibrosis (Ferenbach and Bonventre, 2015). Recent studies have demonstrated that AMPK is closely related to fibrosis-promoting pathways. In a high-fat diet mouse model, 5-aminoimidazole-4-carboxamide ribonucleotide (AICAR, AMPK agonist) decreased both urinary levels of TGF-β1 and mesangial matrix expansion (Declèves et al., 2014). Dugan et al. (2013) verified that in several diabetic nephropathy mouse models, the activation of AMPK dramatically reduced glomerular TGF-β, fibronectin, and collagen accumulation. In addition, several studies have identified the activation of mTOR in diabetic kidney disease (Gödel et al., 2011). mTOR inhibition plays a protective role in diabetic kidney disease, leading to the suppression of kidney fibrosis (Gödel et al., 2011). Despite these studies, the roles of AMPK and mTOR are largely unclear in kidney repair and fibrosis after cisplatin treatment. A comprehensive understanding of the regulation and pathological effects of AMPK and/or mTOR in adaptive and maladaptive kidney repair following cisplatin treatment will facilitate the discovery of genetic and pharmacologic approaches for treating chronic kidney problems in cancer patients after cisplatin chemotherapy.

## Conclusion

Recent studies have established a protective role of autophagy in AKI, including cisplatin nephrotoxicity. AMPK and mTOR are two important regulators of autophagy. While AMPK positively affect autophagy, mTOR is a major negative regulator of autophagy. Currently, the evidence for direct involvement of AMPK and mTOR in autophagy regulation in cisplatin-induced AKI is limited, but several studies have demonstrated that some kidney protective agents may up-regulate autophagy through AMPK and/or mTOR. Obviously, a comprehensive understanding of the regulation of AMPK and mTOR signaling pathways related to autophagy regulation in cisplatin nephrotoxicity may provide further insights into the pathogenesis and suggest new therapeutic strategies.

## Author Contributions

YW, CT, and ZD designed the outline. YW drafted the review. CT and ZD revised the review. JC, ZL, and SS provided suggestions. All authors contributed to the article and approved the submitted version.

## Conflict of Interest

The authors declare that the research was conducted in the absence of any commercial or financial relationships that could be construed as a potential conflict of interest.
